# Trends of risk factors associated with childhood stunting and anaemia in Ghana: evidence from the Demographic Health Survey and Multiple Indicator Cluster Survey (2003–2017)

**DOI:** 10.1017/S1368980023002951

**Published:** 2024-01-23

**Authors:** Christian Sewor, Rajeev Jayalakshmi

**Affiliations:** 1 Department of Public Health and Community Medicine, Central University of Kerala, Kasaragod, KL 671316, India; 2 Public Health Research Group, Department of Biomedical Sciences, University of Cape Coast, Cape Coast, Ghana

**Keywords:** Child, Stunting, Anaemia, Risk factors, Malnutrition

## Abstract

**Objective:**

This study investigated the trend of effect estimates of the key risk factors of childhood stunting and anaemia between 2003 and 2017.

**Design:**

A secondary analysis of the Demographic Health Survey (DHS) and Multiple Indicator Cluster Survey (MICS) data for the Ghanaian population between 2003 and 2017. Associations of selected socio-demographic (child age and gender; maternal age and education), economic (household wealth), environmental, dietary (minimum dietary diversity and iodine use) and health system (place of delivery and vaccination) factors were explored using the Poisson regression model. Trend analysis was explored using a fitted linear regression line on a time series plot.

**Setting:**

Ghana

**Participants:**

Children under 5 years

**Results:**

The results showed a reduction in the prevalence of stunting and anaemia over the 15-year duration. These health outcomes were found to be negatively associated with a wide array of socio-demographic (child age and gender, maternal age and education, residency), economic (household wealth), dietary (iodised salt use) and health service (place of delivery and vaccination) factors; however, the most consistent statistically significant association was observed between child’s age and belonging to the poor wealth quintile.

**Conclusion:**

In order to prevent these indicators of child malnutrition, key consideration must be given to the early developmental stages of life. Child health policies must focus on addressing the key contextual factors of child malnutrition.

Globally, child malnutrition resulting from macro and micronutrient deficiencies is a persisting public health challenge in many low- and middle-income countries. Recent evidence on adverse child health indicators such as stunting and anaemia demonstrates that these conditions remain endemic in many low- and middle-income countries. Childhood stunting as defined by the WHO is having a height-for-age z-score (HAZ) below −2 sd from the median HAZ of the reference population^(1)^. Childhood anaemia amongst children under 5 years of age is given as a Hb concentration less than 110 g/l at sea level^(1)^.

As of 2020, 22·0 % of under 5 years children which translates to about 149·2 million were reported to be stunted globally^(2)^. The WHO report highlighted that although the proportion of stunted children in Africa has decreased over the years, it remains the only region where the absolute number of stunted children is increasing with about 61·4 million children being found stunted^(2)^. Within the West African region, childhood stunting prevalence has been reported to be decreasing. Notwithstanding, the prevalence of stunting which stands at 30·9 % is higher than both Africa’s regional (30·7 %) and global estimates (22·0 %)^(2,3)^.

Similarly, the regional prevalence of anaemia which is 60·2 % is about one and a half times more than the global prevalence (39·8 %)^(4)^. The prevalence estimate for West Africa which is 69·9 % is also higher than both global and regional estimates.

Recent estimates for Ghana, a West African country, indicate that the prevalence of stunting stood at 14·2 % in 2020^(2)^, whereas that of anaemia was 59·5 %^(4)^ making them the child malnutrition indicators, with the largest burden in the country. While both estimates are lower than the respective regional estimates in Africa, the prevalence of anaemia is higher than the global estimate. Over the years, there have been reductions in the prevalence of stunting and anaemia; however, this has occurred at fairly low and inconsistent rates^(3,5,6)^.

These adverse child health outcomes have been found to impair the developmental potential of children and contribute to long-term impairment in cognitive function and adult economic productivity^(7,8)^. Findings from a meta-analysis of data from six African countries highlighted that the risk of death falls by 24 % for a 1 g/dl increase in Hb level^(9)^.

The key reason underlying the continual existence of these adverse child health outcomes in Ghana and other African countries is the prevailing risk factors and the widening gap of the inequalities within the region. Various epidemiological studies reported that childhood stunting and anaemia are significantly associated with socio-economic (household wealth, dietary diversity and minimum acceptable diet), demographic (child age, gender and maternal education), environmental (type of cooking fuel use, sanitation and water source) and health system factors (place of delivery, micronutrient supplementation and child vaccination)^(10,11)^. It is important to note that these factors often exert varying direct and indirect effects on child health outcomes as noted in the UNICEF framework for child undernutrition^(12)^.

While recent child nutrition policies in Ghana have been reported to focus on improving nutrition, water and sanitation practices, social protection, and healthcare access^(13,14)^, these policies are often hampered by endemic inequities, lack of multisectoral coordination, unavailability of funds and in some case a lack policy attention to health indicators^(13)^. This has in turn resulted in an uneven decline in the levels of child malnutrition indicators and the continual proliferation of some of these risk factors. In this regard, there is a need for a comprehensive, integrated, cost-effective and most importantly flexible health policy action to address the health challenges of children. In order to do this, it is crucial to understand the change in the effect estimates of the risk factors of child health outcomes over the years as it enables timely review of policy actions and subsequent modifications focusing on factors that will produce the greatest benefit.

It is thus against this background, that this study sought to identify the key risk factors of childhood stunting and anaemia in Ghana between 2003 and 2017 and the trend of effect estimates associated with these risk factors over the years. By relying on two nationally representative surveys, the Demographic Health Survey (DHS) and the Multiple Indicator Cluster Survey (MICS), this study provides comprehensive evidence on the trend of the effect estimates of each risk factor.

## Methods

### Data source

The study was a quantitative study that involved the secondary analysis of two nationally representative surveys, namely DHS and the MICS conducted in Ghana between 2003 and 2017. For the DHS, datasets of 2003, 2008 and 2014 were analysed, whereas, for MICS, 2006, 2011 and 2017 datasets were used. Cross-sectional study designs which employ nationally representative sample sizes are adopted in both the DHS and MICS.

### Study population and sampling size

The sample units for analysis were children. The analysis was restricted to children under 5 years (0–59 months) for stunting (data were available in DHS and MICS) and children between 6 and 59 months for anaemia (available in only DHS). The DHS programme employs a two-stage stratified sampling, whereas the MICS employs a two-stage cluster sampling design to sample the households before collecting information on women and men (aged 15–49 years) and their children (0–5 years).

### Datasets

For the DHS datasets, the respective Children’s Recode file (KR) which provides information for every child of interviewed women born in the 5 years preceding the survey was used. These datasets contain information related to antenatal care, postnatal care, immunisation and the health of the selected children. The data for the mother of each of these children are included. For MICS datasets, the children’s under 5 years (ch) dataset was merged with the women (wm) and household (hh) datasets using the common identifiers (household line, cluster line and the women’s) in order to include other relevant household and maternal risk factors needed for the analysis as seen in the DHS datasets.

### Research variables

The exposures of interests were socio-demographic (gender and age of the child, maternal age and education, history of illness, low birth weight), dietary (minimum dietary diversity (MDD) and iodised salt in the household), economic (household wealth status), environmental (sanitation, water source and household cooking fuel source) and health service factors (child vaccination, place of delivery and vitamin A supplementation). The outcomes of interest were child anaemia and stunting.

### Data preparation

Since the MICS datasets were in spss format, they were converted into their respective Stata files in IBM SPSS version 26. The required sample weights as per the DHS and MICS guidelines were applied before undertaking the analysis. All missing values and flagged cases were removed before the analysis. Cases that gave unclear responses, such as ‘I don’t know’ and ‘other’ categories, were also dropped wherever applicable. Data available for children who were alive at the time of the survey only were used. The following variables were categorised as follows.

Water sources and sanitation in both the DHS and MICS datasets were classified into improved and unimproved sources based on the WHO/UNICEF criteria^(15)^. Improved sanitation was defined as shared or non-shared facilities that flush/pour-flush to a piped sewer system, septic tank, or pit latrine; ventilated improved pit latrine, pit latrine with a slab, and composting toilet or flush elsewhere. Unimproved sanitation included pit latrines without slab/open pits, bucket toilets, hanging toilets/latrines and others. Improved water sources were the following: piped into dwelling, piped to yard/plot, public tap/standpipe, piped to a neighbour, tube well or borehole, protected well, protected spring, rainwater, tanker truck, cart with a small tank, bottled water, packaged or delivered water. Unimproved water sources were unprotected well, unprotected springs and surface water (river, dam, lake, pond, stream and canal/irrigation channel^(15)^. Household cooking fuel type was categorised as being either clean or polluting. Clean fuels included electricity, liquid petroleum gas, natural gas, biogas and kerosene, whilst polluting fuels included coal/lignite, charcoal, wood, straw/shrubs/grass and animal dung.

MDD was defined by the proportion of children aged 6–23 months who consumed foods from at least four food groups out of the eight referenced food groups within 24 h (four out of eight food groups fed during the day or night preceding the survey). The eight food groups were the following: (1) breastmilk, (2) grains, roots, and tubers, (3) legumes and nuts, (4) dairy products, (5) flesh foods (meat, fish, poultry and liver/organ meats), (6) eggs, (7) vitamin A-rich fruits and vegetables, and (8) other fruits and vegetables^(16)^.

The household wealth index which was initially in quintiles was recategorised into three categories, poor (poorest+poor), middle and rich (rich+richest).

Childhood stunting was defined as having a HAZ below −2 sd below the average on the WHO Child Growth Standards. Childhood anaemia amongst children 6–59 months is given as a Hb concentration less than 110 g/dl.

### Statistical analysis

All statistical analyses were run on Stata version 16 (MP – Parallel Edition). The *svy* command was used in running all the analyses, controlling for both clustering and stratification. Univariate analysis was used to explore the report of the weighted proportions of the health outcomes and the risk factors within each survey year. Simple and multiple Poisson regression models were used to assess the association between the socio-demographic, economic, environmental, and health service factors and maternal and child health indicators across specific survey periods, with the respective prevalence ratios (PR) and 95 % CI generated. A Poisson regression model was used instead of a logistic model because of the high prevalence of both outcomes (stunting and anaemia) within the population. A fitted linear regression line was used to explore the trends of the effect estimates of the significant risk factors.

The risk factors included in the year-wise multiple Poisson regression (adjusted analysis) were selected based on the following criteria:

- Only risk factors which were statistically significant in at least three time points for stunting and two time points for anaemia were included in the multivariate analysis.
- Correlating independent variables were removed. For instance, all household environmental risk factors (sanitation, water source and cooking fuel) were excluded because these were incorporated in the composition of the household wealth^(17,18)^.

In order to further strengthen the robustness of our findings, sensitivity analysis was carried out by pooling all the datasets after which a mixed effect Poisson regression with survey year as a random term was conducted to provide a composite estimate across all survey periods. For the adjusted analysis in the mixed effect Poisson regression, multiple nested models were run in order to ascertain whether the covariates removed in the year-wise adjusted analysis significantly impacted observed relationships. Seeing that the pseudo-R2 and likelihood ratio tests were inappropriate with the *svy* command^(19)^, adjusted Wald test was used to explore the explanatory potential of variables in the regression model. Model fitness in the simple and multiple Poisson regression was explored using the Pearson goodness-of-fit, whereas for the mixed effect models the Akaike and Bayesian information criteria were used (AIC and BIC) to compare model. A *P*-value less than 0·05 was regarded as statistically significant. Percentages were rounded up to one decimal place, whilst PR and CI were reported in two decimal places.

## Results

### Descriptive Pattern of Selected Risk Factors and Child Health Outcomes

Table 1 provides descriptive information on the child outcomes under study and the selected risk factors. About 43–44 % of children interviewed in DHS were aged under 2 years, whereas 24–26 % of them were interviewed in the MICS. The majority of mothers recruited were within the age range of 25–34 years and had completed secondary education or higher.

**Table 1:** Descriptive pattern of selected risk factors and child health outcomes

	2003 (*n* 3467)		2006 (*n* 2806)		2008 (*n* 2627)		2011 (*n* 7750)		2014 (*n* 2710)		2017 (*n* 8879)	
	Weighted		Weighted		Weighted		Weighted		Weighted		Weighted	
Variable	*n*	%	*n*	%	*n*	%	*n*	%	*n*	%	*n*	%
Risk factors												
Dichotomised age category												
Under 2 years	1362	42·5	715·2	25·5	1159	44·1	1543	25·2	1200	44·3	1701	24·0
Two years and above	1841	57·5	2091	74·5	1468	55·9	4581	74·8	1510	55·7	5376	76·0
Gender												
Male	1686	50·5	1789	51·6	1412	51·7	3757	49·8	2822	52·0	4370	49·2
Female	1654	49·5	1678	48·4	1320	48·3	3793	50·2	2608	48·0	4509	50·8
Maternal age (years)												
15–19	113·5	3·4	103·3	3·2	112·1	4·1	232·1	3·3	200·3	3·7	317·8	3·9
20–34	2250	67·4	2210	67·7	1862	68·2	4615	65·5	3641	67·1	5208	63·3
35–49	976	29·2	951·2	29·1	757	27·7	2203	31·2	1589	29·3	2697	32·8
Maternal education												
No education	1339	40·1	1343	38·7	888·1	32·5	2455	32·5	1473	27·1	2431	27·4
Primary	760·9	22·8	752·8	21·7	668·3	24·5	1628	21·6	1084	20·0	1792	20·2
Secondary/higher	1240	37·1	1371	39·6	1175	43·0	3467	45·9	2873	52·9	4657	52·4
Household wealth												
Poor	1604	48·0	1615	46·6	1304	47·7	3281	43·5	2335	43·0	3799	42·8
Middle	655·8	19·6	684·1	19·7	506·5	18·6	1559	20·6	1065	19·6	1771	18·0
Rich	1080	32·3	1168	33·7	921	33·7	2710	35·9	2031	37·4	3308	37·3
Place of residence												
Rural	2225	66·6	2231	64·3	1692	62·0	4267	56·5	2981	54·9	5054	56·9
Urban	1114	33·4	1236	35·7	1039	38·1	3283	43·5	2450	45·1	3825	43·1
Water source												
Improved	2072	62·7	2674	78·0	2268	83·7	6035	79·9	710·8	86·6	7493	84·4
Unimproved	1233	37·3	753·4	22·0	440·3	16·3	1515	20·1	4578	13·4	1382	15·6
Cooking fuel												
Clean fuel	170·6	5·2	244·8	7·1	230·8	8·5	1179	15·6	1072	20·3	1259	14·2
Polluting fuel	3138	94·8	3218	92·9	2479	91·5	6371	84·4	4213	79·7	7611	85·8
Sanitation												
Improved	2287	69·2	1921	55·5	1633	60·5	4285	56·8	3529	67·0	5571	63·0
Unimproved	1019	30·8	1542	44·5	1067	39·5	3262	43·2	1735	33·0	3268	37·0
Low birth weight												
Yes	71·99	2·2	69·1	10·0	117·8	4·3	185·4	8·2	313·2	5·8	308·9	9·7
No	3268	97·8	622·6	90·0	2613	95·7	2072	91·8	5116	94·2	2885	90·3
History of illness												
Yes	1288	39·9	1491	43·1	1080	40·1	2866	38·0	1521	28·4	4345	49·0
No	1945	60·2	1972	57·0	1612	59·9	4680	62·0	3841	71·6	4532	51·1
Minimum dietary diversity (6–23 months)												
Yes	349	32·8			537·5	63·6	969·1	44·5	400	46·0	1086	42·3
No	714·9	67·2			308·3	36·5	1208	55·5	471	54·1	1479	57·7
Household iodised salt												
Yes	1072	34·2	1620	48·1			5196	72·6	3071	62·0	5304	62·4
No	2068	65·9	1749	51·9			1957	27·4	1881	38·0	3191	37·6
Place of delivery												
Health facility	1528	46·0	891·4	44·9	1567	57·7	2789	65·5	3910	73·1	3779	76·5
Home or others	1794	54·0	1093	55·1	1148	42·3	1472	34·6	1438	26·9	1163	23·5
Vitamin A supplement												
Yes	2282	79·6	2281	86·0	1059	70·7	4861	74·3	1475	62·5	1346	73·1
No	581·6	20·31	370·2	14·0	438·4	29·3	1686	25·8	885·2	37·5	496·2	26·9
Taken any vaccine												
Yes	685·1	74·4	788·7	88·3	583·4	84·4	1284	92·7	1013	88·8	431·1	63·0
No	235·5	25·6	104·1	11·7	107·6	15·6	101·1	7·3	127·5	11·2	253·3	37·0
Health outcomes												
Childhood stunting (0–59 months)												
Stunted	861·4	29·4	893·8	27·9	639	27·5	1669	22·8	472·6	17·9	1514	17·5
Nourished	2066	70·6	2306	72·1	1687	72·5	5669	77·3	2164	82·1	7125	82·5
Childhood anaemia (6–59 months)												
Anaemic	2071	76·5			1660	78·4			1544	66·8		
Not anaemic	634·9	23·5			456·8	21·6			767·5	33·2		

Data for minimum dietary diversity and household iodised salt usage were unavailable in 2006 and 2008, respectively.

Most of the mothers and their children interviewed resided in poor households and rural areas. Concerning household environmental conditions, a majority of households across all the survey periods had improved water and sanitation sources and used polluting fuel.

The prevalence of low birth weight and history of illness amongst the children was observed to have shown an increasing trend. The proportion of children who were found to have met the MDD (6 and 23 months) was highest in 2008. An increasing number of households were observed to have been consuming iodised salt across the survey periods.

There was an increase in the proportion of hospital delivery across survey periods, and the highest prevalence of vitamin A supplementation was reported in 2006. Also, the highest proportion of children who reported to have received any vaccination was 93 % (in 2008)

The prevalence of stunting was observed to have decreased across the survey period with the highest estimate of 29·4 % recorded in 2003 and the lowest estimate of 17·5 % recorded in 2017. The prevalence of anaemia amongst children aged 6–59 months, on the other hand, was the highest at 78·4 % in 2008 and the lowest at 66·8 % recorded in 2014.

### Association and trends of risk factors associated with child health outcomes

**Fig. 1:**
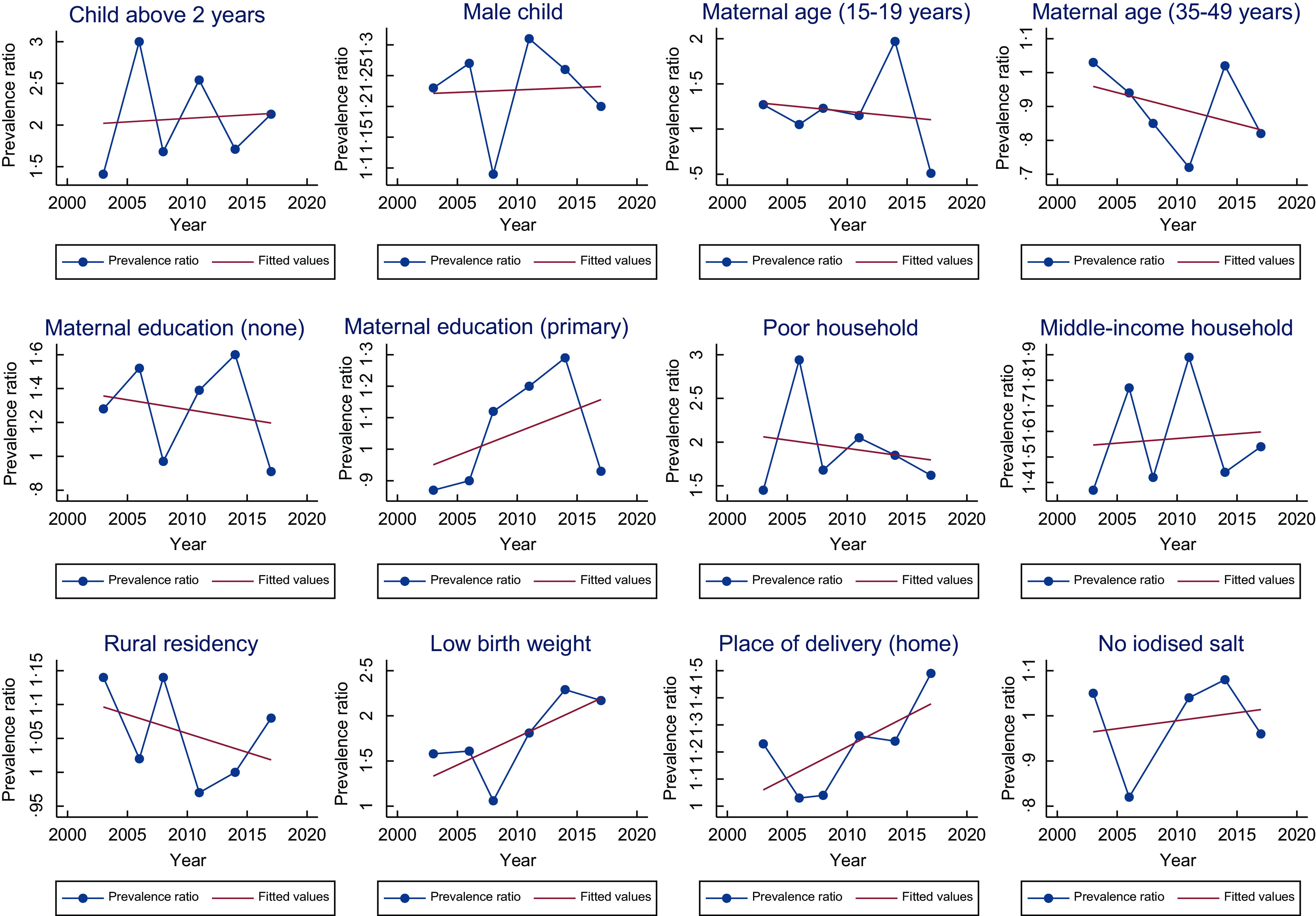
Trend of prevalence ratio of the risk factors associated with childhood stunting

From the results, the key risk factors associated with stunting (Tables 2 and 4 ) were child age (above two years) and gender (male), maternal education (no education), household wealth (poor), low birth weight, and place of delivery (delivering at home or other places besides the hospital). Consistently, across the majority of the survey years, stunting was more likely amongst children above 2 years, low birth weight children, and children residing in poor households. On the contrary in 2017, we observed that children born to teenage mothers (15–19 years) were less likely to be stunted children than early adult mothers (20–34 years) (PR = 0·51, 95 % CI 0·28, 0·94). The PR of child age and gender, mothers with primary education, middle-income households, low birth weight, place of delivery (home) and use of iodised salt in homes showed an increasing trend, whereas the remaining showed a decreasing trend (Fig. 1).

**Table 2:** Crude prevalence ratios of risk factors associated with stunting

Variable	2003		2006		2008		2011		2014		2017	
Risk factors	PR	95 CI %	PR	95 CI %	PR	95 CI %	PR	95 CI %	PR	95 CI %	PR	95 CI %
Dichotomised age												
Under 2 years	1		1		1		1		1		1	
Above 2 years	1·42	1·25, 1·61***	2·85	2·24, 3·64***	1·65	1·42, 1·92***	2·75	2·19, 3·45***	1·78	1·43, 2·21***	2·44	1·94, 3·07***
Gender												
Male	1·22	1·08, 1·38**	1·14	1·00, 1·30*	1·12	0·96, 1·30	1·25	1·10, 1·41***	1·24	1·05, 1·46*	1·25	1·06, 1·48**
Female	1		1		1		1		1		1	
Maternal age (years)												
15–19	1·06	0·75, 1·49	1·07	0·73, 1·58	1·07	0·75, 1·53	1·07	0·79, 1·45	1·72	1·24, 2·39**	0·69	0·47, 1·01
20–34	1·00		1		1		1		1		1	
35–49	1·14	1·01, 1·29*	1·14	1·00, 1·30*	0·94	0·79, 1·11	1·07	0·93, 1·23	1·11	0·91, 1·35	0·85	0·74, 0·99*
Maternal education												
No education	1·60	1·38, 1·86***	1·73	1·50, 2·00***	1·26	1·05, 1·51*	1·73	1·50, 1·98***	2·05	1·67, 2·52***	1·68	1·41, 2·00***
Primary	1·01	0·82, 1·24	1·24	1·02, 1·50*	1·34	1·10, 1·64**	1·49	1·23, 1·80***	1·56	1·21, 2·00**	1·21	1·00, 1·47
Secondary/higher	1		1		1		1		1		1	
Household wealth												
Poor	1·99	1·68, 2·36***	2·48	2·05, 3·01***	1·86	1·52, 2·29***	2·19	1·80, 2·66***	2·39	1·78, 3·22***	1·85	1·57, 2·17***
Middle	1·64	1·31, 2·04***	1·88	1·52, 2·34***	1·54	1·18, 2·02**	1·66	1·30, 2·13***	1·62	1·14, 2·32**	1·38	1·12, 1·69**
Rich	1		1		1		1		1		1	
Place of residence												
Rural	1·71	1·44, 2·02***	1·95	1·60, 2·37**	1·58	1·28, 1·94***	1·45	1·24, 1·71***	1·65	1·32, 2·06***	1·47	1·22, 1·77***
Urban	1		1		1				1			
Water source												
Unimproved	1·30	1·14, 1·48***	1·34	1·15, 1·57***	1·26	1·05, 1·51*	1·55	1·35, 1·78***	1·28	1·0, 1·64	1·66	1·38, 1·98***
Improved	1		1		1		1		1		1	
Cooking fuel												
Polluting fuel	3·42	1·65, 7·10**	3·77	2·00, 7·12***	2·12	1·33, 3·38**	2·27	1·65, 3·12***	2·62	1·68, 4·08***	2·16	1·63, 2·85***
Clean fuel	1		1		1		1		1		1	
Sanitation												
Unimproved	1·55	1·37, 1·74***	1·46	1·26, 1·70***	1·33	1·14, 1·56***	1·61	1·40, 1·84***	1·38	1·12, 1·71**	1·42	1·22, 1·66***
Improved	1		1		1		1		1		1	
Low birth weight												
No	1		1		1		1		1		1	
Yes	1·19	0·81, 1·75	2·04	1·35, 3·10**	0·88	0·62, 1·25	1·64	1·11, 2·42*	1·51	1·11, 2·06**	1·96	1·49, 2·58***
History of illness												
No	1		1		1		1		1		1	
Yes	0·90	0·80, 1·01	1·03	0·91, 1·15	1·10	0·95, 1·27	1·17	1·03, 1·32*	0·91	0·74, 1·14	1·29	1·14, 1·46***
Minimum dietary diversity												
No	1·11	0·86, 1·44	–		0·87	0·64, 1·16	0·93	0·74, 1·17	1·34	0·91, 1·95	0·78	0·62, 0·99*
Yes	1		–		1		1		1		1	
Place of delivery												
Home or others	1·61	1·41, 1·84***	1·71	1·40, 2·08***	1·31	1·12, 1·54**	1·68	1·42, 1·98***	1·73	1·43, 2·09***	1·87	1·56, 2·45***
Health facility	1		1		1		1		1		1	
Vitamin A dose												
No	0·99	0·84, 1·16	1·05	0·84, 1·30	1·23	1·01, 1·49*	0·98	0·84, 1·14	1·38	1·13, 1·68**	0·99	0·72, 1·36
Yes	1		1		1		1		1		1	
Iodised salt												
Yes	1		1		–		1		1		1	
No	1·31	1·14, 1·52***	1·19	1·03, 1·37*	–		1·26	1·11, 1·45**	1·27	1·00, 1·62	1·26	1·06, 1·50**
Taken any vaccine†												
Yes	–		1		1		1		1		1	
No	–		1·39	0·92, 2·11	0·56	0·35, 0·92*	0·95	0·58, 1·56	1·10	0·54, 2·24	1·04	0·65, 1·66

PR, prevalence ratio.

* *P*-value < 0·05.

** *P*-value < 0·01.

*** *P*-value < 0·001.

† Model for 2003 failed to converge.

**Table 3:** Crude prevalence ratios of risk factors associated with anaemia

	2003		2008		2014	
Risk factors	PR	95 CI %	PR	95 CI %	PR	95 CI %
Dichotomised age						
Under 2 years	1·11	1·06, 1·16***	1·14	1·08, 1·20***	1·27	1·18, 1·36***
Above 2 years	1		1		1	
Gender						
Male	0·99	0·95, 1·04	1·12	0·96, 1·30	1·0	0·93, 1·07
Female	1		1		1	
Maternal age (years)						
15–19	1·12	1·03, 1·23*	1·13	1·03, 1·23**	1·17	0·94, 1·44
20–34	1·00		1		1	
35–49	0·96	0·92, 1·02	0·97	0·91, 1·03	1·00	0·92, 1·09
Maternal education						
No education	1·17	1·11, 1·24***	1·14	1·07, 1·22***	1·36	1·26, 1·48***
Primary	1·12	1·05, 1·19**	1·10	1·03, 1·18**	1·16	1·05, 1·29**
Secondary/higher	1		1		1	
Household wealth						
Poor	1·26	1·17, 1·35***	1·29	1·20, 1·40***	1·45	1·31, 1·59***
Middle	1·23	1·14, 1·34***	1·22	1·11, 1·34***	1·16	1·02, 1·32*
Rich	1		1		1	
Place of residence						
Rural	1·18	1·10, 1·26***	1·25	1·17, 1·35***	1·23	1·12, 1·35***
Urban	1		1		1	
Water source						
Unimproved	1·08	1·04, 1·14**	1·11	1·05, 1·17**	1·18	1·08, 1·29***
Improved	1		1		1	
Cooking fuel						
Polluting fuel	1·48	1·22, 1·81***	1·51	1·25, 1·83***	1·47	1·26, 1·71***
Clean fuel	1		1		1	
Sanitation						
Unimproved	1·09	1·04, 1·15***	1·14	1·08, 1·21***	1·28	1·19, 1·37***
Improved	1		1		1	
Low birth weight						
No	1		1		1	
Yes	1·03	0·89, 1·19	0·90	0·77, 1·05	0·79	0·66, 0·94**
History of illness						
No	1		1		1	
Yes	1·01	0·97, 1·07	1·12	1·06, 1·17***	1·12	1·03, 1·22**
Minimum dietary diversity (6–23 months)						
No	1·02	0·96, 1·09	1·02	0·96, 1·09	1·10	0·99, 1·22
Yes	1		1		1	
Place of delivery						
Home or others	1·17	1·12, 1·23***	1·15	1·10, 1·21***	1·26	1·18, 1·34***
Health facility	1		1		1	
Vitamin A dose						
No	1·01	0·96, 1·08	1·01	0·94, 1·07	1·02	0·94, 1·10
Yes	1		1		1	
Iodised salt						
No	1·11	1·05, 1·17***	–		1·15	1·06, 1·24**
Yes	1		–		1	
Taken any vaccine						
Yes	–		1		1	
No	–		1·13	0·98, 1·31	1·38	1·17, 1·62***

PR, prevalence ratio.

* *P*-value < 0·05.

** *P*-value < 0·01.

*** *P*-value < 0·001.

**Fig. 2:**
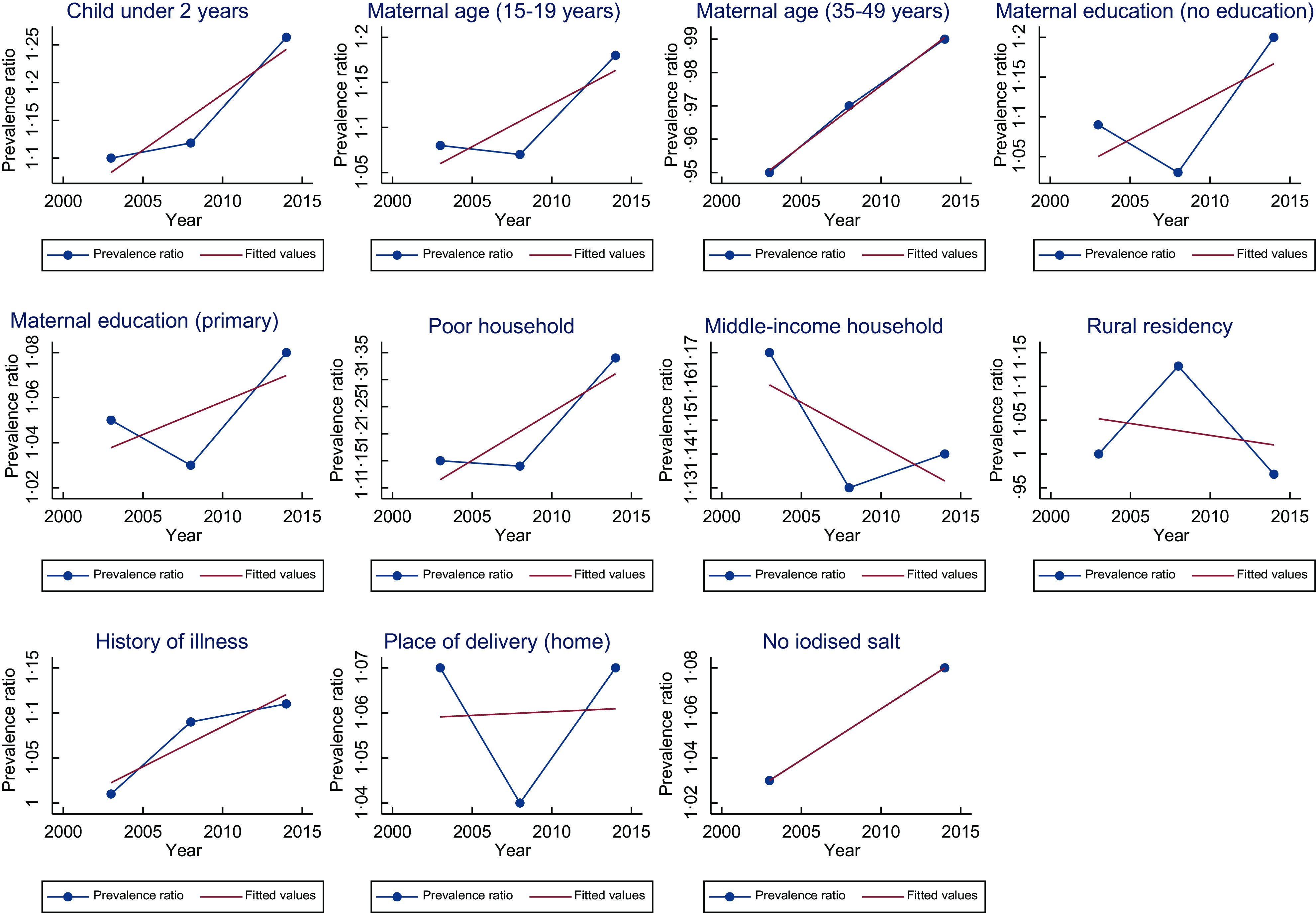
Trend of prevalence ratio of the risk factors associated with childhood anaemia

The key risk factors associated with anaemia (Tables 3 and 5) were child age (under 2 years), maternal education (no education), household wealth and place of delivery, history of illness, place of residence, and household iodised salt use. Consistently, across the majority of the survey years, childhood anaemia was more likely amongst children under 2 years, those born to mothers with no education, children residing in poor households and children with prior history of illness. The PR of all risk factors associated with anaemia showed an increasing trend in all risk factors except middle-income households and rural households (Fig. 2).

**Table 4:** Adjusted prevalence ratios of risk factors associated with stunting

	2003 (*n* 583)		2006 (*n* 2760)		2008 (*n* 2314)		2011 (*n* 1861)		2014 (*n* 2354)		2017 (*n* 2644)	
Risk factors	PR	95 CI %	PR	95 CI %	PR	95 CI %	PR	95 CI %	PR	95 CI %	PR	95 CI %
Dichotomised age												
Under 2 years	1		1		1		1		1		1	
Above 2 years	1·41	1·23, 1·62***	3·00	1·92, 4·68***	1·68	1·44, 1·96***	2·54	1·74, 3·70***	1·71	1·37, 2·15***	2·13	1·60, 2·83***
Gender												
Male	1·23	1·09, 1·39**	1·27	0·90, 1·79	1·09	0·94, 1·26	1·31	0·99, 1·72	1·26	1·07, 1·47**	1·20	0·92, 1·56
Female	1		1		1		1		1		1	
Maternal age (years)												
15–19	1·27	0·90, 1·78	1·05	0·39, 2·84	1·23	0·87, 1·73	1·15	0·69, 1·91	1·97	1·35, 2·86***	0·51	0·28, 0·94*
20–34	1		1		1		1		1		1	
35–49	1·03	0·90, 1·18	0·94	0·62, 1·43	0·85	0·72, 1·02	0·72	0·51, 1·01	1·02	0·83, 1·25	0·82	0·58, 1·15
Maternal education												
No education	1·28	1·08, 1·51**	1·52	0·89, 2·59	0·97	0·80, 1·18	1·39	0·93, 2·10	1·60	1·20, 2·13**	0·91	0·63, 1·31
Primary	0·87	0·70, 1·08	0·90	0·52, 1·55	1·12	0·91, 1·37	1·20	0·80, 1·78	1·29	0·98, 1·72	0·93	0·68, 1·29
Secondary/higher	1		1		1		1		1		1	
Household wealth												
Poor	1·45	1·16, 1·82**	2·94	1·62, 5·34***	1·68	1·27, 2·21***	2·05	1·25, 3·35**	1·85	1·19, 2·88**	1·62	1·08, 2·43*
Middle	1·37	1·08, 1·75**	1·77	0·99, 3·18	1·42	1·09, 1·86*	1·89	1·20, 2·96**	1·44	0·97, 2·14	1·54	1·06, 2·24*
Rich	1		1		1		1		1		1	
Place of residence												
Rural	1·14	0·92, 1·41	1·02	0·66, 1·58	1·14	0·86, 1·51	0·97	0·67, 1·40	1·00	0·73, 1·36	1·08	0·75, 1·55
Urban	1		1		1		1		1		1	
Low birth weight												
Yes	1·58	1·06, 2·37*	1·61	1·04, 2·50*	1·06	0·74, 1·51	1·81	1·23, 2·69**	2·29	1·70, 3·07***	2·17	1·60, 2·95***
No	1		1		1		1		1		1	
Place of delivery												
Home or others	1·23	1·04, 1·46*	1·03	0·72, 1·46	1·04	0·86, 1·25	1·26	0·70, 2·27	1·24	1·04, 1·49*	1·49	0·77, 2·86
Health facility	1		1		1		1		1		1	
Iodised salt												
No	1·05	0·91, 1·21	0·82	0·57, 1·19	–		1·04	0·77, 1·42	1·08	0·86, 1·36	0·96	0·70, 1·31
Yes	1		1		–		1		1		1	
Model fitness details												
Adjusted Wald test	F(12, 372) = 14·27 Prob > F= < 0·001		F(12, 172) = 8·69 Prob > F = < 0·001		F(11, 374) = 8·93 Prob > F= < 0·001		F(12, 523) = 8·23 Prob > F = < 0·001		F(12, 384) = 10·78 Prob > F= < 0·001		F(12, 578) = 8·33 Prob > F = < 0·001	
Pearson goodness-of-fit	Pearson goodness-of-fit = 1944·19 Prob > chi2(2912) = 1·00		Pearson goodness-of-fit = 475·18 Prob > chi2(516) = 0·90		Pearson goodness-of-fit = 1679·76 Prob > chi2(2352) = 1·00		Pearson goodness-of-fit = 1510·65 Prob > chi2(1401) = 0·02		Pearson goodness-of-fit= 1909·39 Prob > chi2(2465)= 1·00		Pearson goodness-of-fit = 2242·81 Prob > chi2(2652)= 1·00	

PR, prevalence ratio.

* *P*-value < 0·05.

** *P*-value < 0·01.

*** *P*-value < 0·001.

**Table 5:** Adjusted prevalence ratios of risk factors associated with anaemia

	2003 (*n* 2535)		2008 (*n* 2098)		2014 (*n* 2079)	
Risk factors	PR	95 CI %	PR	95 CI %	PR	95 CI %
Dichotomised age						
Under 2 years	1·10	1·05, 1·15***	1·12	1·06, 1·17***	1·26	1·18, 1·35***
Above 2 years	1		1		1	
Maternal age (years)						
15–19	1·08	0·99, 1·18	1·07	0·98, 1·17	1·18	1·00, 1·40*
20–34	1		1		1	
35–49	0·95	0·90, 1·01	0·97	0·92, 1·03	0·99	0·91, 1·08
Maternal education						
No education	1·09	1·02, 1·16***	1·03	0·96, 1·10	1·20	1·09, 1·33***
Primary	1·05	0·98, 1·13	1·03	0·96, 1·09	1·08	0·97, 1·19
Secondary/higher	1		1		1	
Household wealth						
Poor	1·15	1·04, 1·27**	1·14	1·05, 1·24**	1·34	1·13, 1·59**
Middle	1·17	1·07, 1·29**	1·13	1·03, 1·24**	1·14	0·96, 1·34
Rich	1		1		1	
Place of residence						
Rural	1·00	0·91, 1·10	1·13	1·04, 1·23**	0·97	0·86, 1·09
Urban	1		1		1	
History of illness						
Yes	1·01	0·96, 1·07	1·09	1·04, 1·14**	1·11	1·03, 1·20**
No	1		1		1	
Place of delivery						
Home or others	1·07	1·01, 1·12*	1·04	0·99, 1·09	1·07	1·00, 1·14
Health facility	1		1		1	
Iodised salt						
No	1·03	0·97, 1·08	–		1·08	1·00, 1·17*
Yes	1		–		1	
Model fitness details						
Adjusted Wald test	F(11, 374) = 7·30 Prob > F = <0·001		F(11, 374) = 8·93 Prob > F = < 0·001		F(11, 385) = 16·91 Prob > F = < 0·001	
Pearson goodness-of-fit	Pearson goodness-of-fit = 592·92 Prob > chi2(2690) = 1·00		Pearson goodness-of-fit = 452·63 Prob > chi2(2134) = 1·00		Pearson goodness-of-fit = 691·07 Prob > chi2(2173) = 1·00	

PR, prevalence ratio.

* *P*-value < 0·05.

** *P*-value < 0·01.

*** *P*-value < 0·001.

### Sensitivity analysis

The results for the mixed model (Tables 6 and 7) that contained all the risk factors were found to have the smallest AIC. These results did not significantly differ from the main analysis, with the exception of MDD and childhood vaccination which were found as potential additional risk factors for stunting (Table 6) and anaemia (Table 7), respectively.

**Table 6:** Prevalence ratios of risk factors associated with stunting (mixed effect Poisson regression)

Risk factors	cPR	95 CI %	aPR	95 CI %^1^	aPR	95 CI %^2^	aPR	95 CI %^3^	aPR	95 CI %^4^	aPR	95 CI %^5^	aPR	95 CI %^6^
Dichotomised age														
Under 2 years	1		1		1		1		1		1		1	
Above2 years	2·02	1·57, 2·59***	1·58	1·30, 1·91***	1·57	1·28, 1·92***	1·57	1·30, 1·91***	1·50	1·29, 1·74***	1·49	1·27, 1·74***	1·49	1·29, 1·73***
Gender														
Male	1·21 (	1·17, 1·26***	1·23	1·19, 1·27***	1·23	1·19, 1·26***	1·23	1·20, 1·27***	1·24	1·22, 1·25***	1·23	1·22, 1·24***	1·238	1·233, 1·243***
Female	1		1		1		1		1		1		1	
Maternal age (years)														
15–19	1·01	0·80, 1·28	1·56	1·08, 2·24*	1·58	1·09, 2·29*	1·59	1·06, 2·39*	–		–		–	
20–34	1		1		1		1		–		–		–	
35–49	1·02	0·91, 1·14	1·02	1·01, 1·04*	1·019	1·014, 1·024***	1·03	1·02, 1·05***	–		–		–	
Maternal education														
No education	1·67	1·55, 1·80***	1·34	1·01, 1·76*	1·42	1·17, 1·73***	1·40	1·00, 1·95	1·30	1·00, 1·69	1·38	1·15, 1·65***	1·36	0·98, 1·88
Primary	1·30	1·16, 1·47***	1·00	0·71, 1·40	1·05	0·72, 1·52	1·02	0·70, 1·50	0·98	0·68, 1·41	1·03	0·70, 1·52	1·01	0·67, 1·51
Secondary/higher	1				1		1				1			
Household wealth														
Poor	2·08	1·88, 2·30***	1·40	1·19, 1·64***	1·60	1·32, 1·93***	–		1·41	1·12, 1·76**	1·62	1·24, 2·11***	–	
Middle	1·59	1·44, 1·76***	1·24	1·18, 1·30***	1·42	1·38, 1·46***	–		1·250	1·246, 1·253***	1·43	1·32, 1·56***	–	
Rich	1		1		1				1		1			
Place of residence														
Rural	1·57	1·44, 1·72***	1·06	0·97, 1·16	1·07	0·96, 1·21	1·23	1·17, 1·29***	1·06	0·94, 1·20	1·08	0·93, 1·25	1·24	1·17, 1·30***
Urban	1		1											
Water source														
Unimproved	1·45	1·31, 1·59***	0·96	0·86, 1·09	–		1·00	0·91, 1·10	0·96	0·86, 1·07	–		1·00	0·92, 1·07
Improved	1		1		–		1		1		–			
Cooking fuel														
Polluting fuel	2·39	2·13, 2·70***	2·24	1·62, 3·09***	–		2·45	1·84, 3·27***	2·29	1·70, 3·10***	–		2·52	1·96, 3·22***
Clean fuel	1		1		–		1		1		–			
Sanitation														
Unimproved	1·48	1·40, 1·57***	1·06	0·78, 1·44	–		1·10	0·84, 1·44	1·06	0·79, 1·43	–		1·10	0·85, 1·42
Improved	1		1		–		1		1					
Low birth weight														
Yes	1·51	1·19, 1·92**	2·00	1·47, 2·72***	1·95	1·40, 2·70***	1·98	1·49, 2·63***	2·00	1·45, 2·77***	1·95	1·38, 2·75***	1·98	1·48, 2·66***
No	1		1		1		1		1		1		1	
History of illness														
Yes	1·11	0·99, 1·24	0·96	0·87, 1·07	0·97	0·88, 1·07	0·96	0·87, 1·07	–		–		–	
No	1		1		1		1		–		–		–	
Minimum dietary diversity														
No	0·93	0·81, 1·08	0·85	0·76, 0·94**	0·86	0·80, 0·91***	0·85	0·77, 0·95**	–		–		–	
Yes	1		1		1		1		–				–	
Institutional delivery														
Home or others	1·65	1·49, 1·82***	1·249	1·247, 1·250***	1·24	1·23, 1·26***	1·284	1·281, 1·288***	1·25	1·22, 1·27***	1·24	1·23, 1·25	1·28	1·26, 1·31***
Health facility	1		1		1		1		1		1			
Vitamin A dose														
No	1·06	0·95, 1·19	0·94	0·74, 1·20	0·93	0·74, 1·18	0·94	0·74, 1·20	–		–		–	
Yes	1		1		1		1		–		–		–	
Iodised salt														
No	1·26	1·23, 1·29***	1·01	0·97, 1·06	1·04	1·03, 1·06***	1·02	0·98, 1·07	1·03	1·00, 1·06*	1·06	1·05, 1·06***	1·04	1·01, 1·07**
Yes	1		1		1		1		1		1		1	
Taken any vaccine														
No	1·15	1·02, 1·30*	1·02	0·98, 1·06	1·03	1·00, 1·06	1·04	1·00, 1·07	1·02	0·97, 1·08	1·03	0·98, 1·07	1·03	0·98, 1·09
Yes	1		1		1		1		1		1		1	
Model fitness			Adjusted Wald test: *P*-value > 0·001 AIC: 5548·672 BIC: 5561·824		Adjusted Wald test: *P*-value > 0·001 AIC: 5590·577 BIC: 5603·737		Adjusted Wald test: *P*-value = 0·03 AIC: 5555·583 BIC: 5562·159		Adjusted Wald test: *P*-value > 0·001 AIC: 5649·485 BIC: 5662·667		Adjusted Wald test: *P*-value > 0·001 AIC: 5693·811 BIC: 5707		Adjusted Wald test: *P*-value = 0·973 AIC: 5658·895 BIC: 5672·076	

cPR, crude prevalence ratio; aPR, adjusted prevalence ratio; AIC, Akaike information criterion, BIC; Bayesian information criterion.

Model 1: Multiple regression model which included all risk factors.

Model 2: Multiple regression model which included all risk factors except household environmental conditions (cooking fuel, sanitation and water source).

Model 3: Multiple regression model which includes all risk factors except household wealth.

Model 4: Multiple regression model which includes only significant risk factors.

Model 5: Multiple regression model which includes only significant risk factors in unadjusted analysis and household environmental conditions.

Model 6: Multiple regression model which includes only significant risk factors in unadjusted analysis and household wealth.

* *P*-value < 0·05.

** *P*-value < 0·01.

*** *P*-value < 0·001.

**Table 7:** Prevalence ratios of risk factors associated with anaemia (mixed effect Poisson regression)

Risk factors	cPR	95 CI %	aPR	95 CI %^1^	aPR	95 CI %^2^	aPR	95 CI %^3^	aPR	95 CI %^4^	aPR	95 CI %^5^	aPR	95 CI %^6^
Dichotomised age														
Under 2 years	1·16	1·08, 1·26***	1·16	1·04, 1·30**	1·16	1·04, 1·30**	1·16	1·04, 1·30*	1·16	1·04, 1·30**	1·16	1·04, 1·30*	1·16	1·04, 1·31*
Above 2 years	1		1		1		1		1		1		1	
Gender														
Male	1·01	0·98, 1·03	1·02	0·97, 1·06	1·01	0·97, 1·06	1·02	0·97, 1·06	–		–		–	
Female	1		1		1		1							
Maternal age (years)														
15–19	1·14	1·11, 1·16***	1·11	1·02, 1·22*	1·12	1·02, 1·23*	1·12	1·03, 1·23*	1·11	1·03, 1·19**	1·12	1·03, 1·22**	1·12	1·03, 1·22**
20–34	1		1		1		1		1		1		1	
35–49	0·98	0·95, 1·00*	0·98	0·95, 1·01	0·97	0·94, 1·01	0·98	0·95, 1·02	0·98	0·94, 1·01	0·98	0·94, 1·02	0·97	0·94, 1·01
Maternal education														
No education	1·22	1·10, 1·35***	1·12	1·02, 1·22*	1·14	1·02, 1·26*	1·14	1·01, 1·28*	1·12	1·02, 1·23*	1·14	1·01, 1·28*	1·14	1·02, 1·26*
Primary	1·13	1·09, 1·17***	1·05	1·01, 1·10*	1·08	1·02, 1·14**	1·07	1·01, 1·13*	1·05	1·01, 1·10*	1·06	1·01, 1·12*	1·08	1·02, 1·13**
Secondary/higher	1		1		1		1		1		1		1	
Household wealth														
Poor	1·32	1·22, 1·43***	1·14	1·05, 1·25**	1·21	1·06, 1·38**	–		1·14	1·04, 1·25**			1·21	1·06, 1·39**
Middle	1·21	1·17, 1·25***	1·09	1·01, 1·17*	1·15	1·11, 1·19***	–		1·09	1·02, 1·16*			1·15	1·12, 1·18***
Rich	1		1		1				1				1	
Place of residence														
Rural	1·22	1·17, 1·27***	0·99	0·97, 1·01	0·99	0·96, 1·02	1·052	1·051, 1·053***	0·99	0·97, 1·01	1·052	1·050, 1·054***	0·99	0·97, 1·02
Urban	1		1		1		1		1		1		1	
Water source														
Unimproved	1·11	1·06, 1·16***	1·005	1·000, 1·010*	–		1·02	0·997, 1·036	1·005	1·001, 1·009*	1·02	1·00, 1·03	–	
Improved	1		1		–		1		1		1			
Cooking fuel														
Polluting fuel	1·51	1·46, 1·55***	1·29	1·18, 1·42***	–		1·34	1·23, 1·45***	1·29	1·18, 1·42***	1·34	1·24, 1·45***	–	
Clean fuel	1		1		–		1		1		1			
Sanitation														
Unimproved	1·16	1·06, 1·27**	0·9977	0·9966, 0·9997*	–		1·02	0·99, 1·05	0·997	0·996, 0·999**	1·02	0·99, 1·05	–	
Improved	1		1		–		1		1		1		–	
Low birth weight														
Yes	0·87	0·75, 1·01	0·98	0·81, 1·17	0·96	0·79, 1·16	0·97	0·81, 1·17	–		–		–	
No	1		1		1		1		–		–		–	
History of illness														
Yes	1·08	1·01, 1·16*	1·05	0·98, 1·13	1·06	0·97, 1·14	1·05	0·98, 1·12	1·05	0·97, 1·13	1·05	0·97, 1·13	1·06	0·97, 1·15
No	1		1		1		1		1		1		1	
Minimum dietary diversity														
No	1·04	0·99, 1·10	0·99	0·98, 1·01	1·00	0·97, 1·02	1·00	0·99, 1·01	–		–		–	
Yes	1		1		1		1		–		–		–	
Institutional delivery														
Home or others	1·19	1·14, 1·25***	1·07	1·05, 1·09***	1·07	1·06, 1·08***	1·08	1·06, 1·10***	1·07	1·05, 1·08***	1·08	1·07, 1·09***	1·07	1·06, 1·08***
Health facility	1		1		1		1		1		1		1	
Vitamin A dose														
No	1·011	1·010, 1·013***	0·97	0·93, 1·02	0·970	0·942, 0·998*	0·98	0·93, 1·02	0·97	0·93, 1·02	0·98	0·93, 1·02	0·97	0·940, 0·999*
Yes	1		1		1		1		1		1		1	
Iodised salt														
No	1·13	1·09, 1·17***	1·04	0·99, 1·09	1·05	1·00, 1·11	1·04	0·99, 1·09	1·04	0·99, 1·09	1·04	0·99, 1·09	1·05	1·00, 1·11
Yes	1		1		1		1		1		1		1	
Taken any vaccine														
No	1·13	1·11, 1·15***	1·05	1·02, 1·08**	1·06	1·02, 1·09**	1·06	1·03, 1·09***	1·05	1·02, 1·09**	1·06	1·03, 1·09***	1·06	1·02, 1·09**
Yes	1		1		1		1		1		1		1	
Model fitness			Adjusted Wald test: *P*-value > 0·001 AIC: 8588·909 BIC: 8601·87		Adjusted Wald test: *P*-value = 0·700 AIC: 8622·912 BIC: 8629·396		Adjusted Wald test: *P*-value > 0·001 AIC: 8593·206 BIC: 8606·166		Adjusted Wald test: *P*-value > 0·001 AIC: 8589·192 BIC: 8602·153		Adjusted Wald test: *P*-value = 0·04 AIC: 8591·505 BIC: 8597·985		Adjusted Wald test: *P*-value = 0·006 AIC: 8625·285 BIC 8638·253	

cPR, crude prevalence ratio; aPR, adjusted prevalence ratio; AIC, Akaike information criterion; BIC, Bayesian information criterion.

^1^ Model 1: Multiple regression model which includes all risk factors.

^2^ Model 2: Multiple regression model which includes all risk factors except household environmental conditions (cooking fuel, sanitation and water source).

^3^ Model 3: Multiple regression model which includes all risk factors except household wealth.

^4^ Model 4: Multiple regression model which includes only significant risk factors.

^5^ Model 5: Multiple regression model which includes only significant risk factors in unadjusted analysis and household environmental conditions.

^6^ Model 6: Multiple regression model which includes only significant risk factors in unadjusted analysis and household wealth.

* *P*-value < 0·05.

** *P*-value < 0·01.

*** *P*-value < 0·001.

## Discussion

We observed that childhood stunting and anaemia were significantly negatively associated with various socio-demographic (child-level age and gender, maternal age and education, residence), economic (household wealth), nutrition (iodised salt use) and health service (place of delivery and vaccination) factors. The most consistent among these across all survey periods were the child’s age and living in a poor household.

### Risk factors of stunting

Epidemiological studies conducted have provided indicative evidence that corroborates the risk factors of stunting noted in this study. For instance, concerning child-level factors such as age and gender, other epidemiological studies have reported similar findings. Jonah *et al.* ^(20)^ and Keino *et al.* ^(21)^ reported that childhood stunting in Africa seems to be higher amongst male children as was observed in this present study. A possible reason was highlighted by Thompson^(22)^, who noted that gender differences in growth trajectories which start during the early developmental stages of life predispose boys to a greater risk of infection and undernutrition.

Maternal risk factors such as maternal age and education were identified as key risk factors. Generally, educated women are more likely to have a direct influence on the dietary choices of their children, thereby reducing the risk of undernutrition. Concerning maternal age, younger mothers are more likely to give birth to children with low birth weight who are likely to grow to be undernourished^(23)^. Reviews by Akombi *et al.* ^(24)^ and Obasohan *et al.* ^(25)^ on SSA have all reported that maternal age is associated with childhood stunting status.

Furthermore, adverse birth outcomes such as low birth weight have been identified to be associated with child stunting status. As was reported in a review by Quamme and Iversen^(26)^, childhood stunting in SSA is common amongst children born with low birth weight.

Seeing that household wealth has been reported to be a significant predictor of health status, with poor households being reported to have poor nutritional, economic, environmental and health status^(27)^. It thus comes as no surprise that it was a significant risk factor for stunting as has been reported^(25,28)^.

### Risk factors associated with anaemia

In a study by Shenton *et al.* ^(29)^, various child factors (age and fever) household level (water source, toilet and wealth) maternal factors (age, education and anaemia status), family-related factors (number of wives and children) religion and ethnicity were noted to be significantly associated with anaemia between 2003 and 2014 in Ghana. The study found child age, history of illness (fever) and maternal anaemia status to be the most consistent risk factors for childhood anaemia in Ghana. Also, a review by Obasohan *et al.* ^(30)^ reported anaemia in SSA to be significantly associated with child age and gender, birth order, maternal education, age, and anaemia, the occurrence of co-morbidities (such as fever, diarrhoea and acute respiratory infection), malnutrition, household wealth and place of residence. From these sets identified, five of the risk factors were identified in this study.

Childhood anaemia seems to disproportionately affect younger children with a relatively high prevalence reported amongst children below 2 years^(31)^. A plausible reason underlying this observation could be the fact that the period from prenatal life to the first 24 months after birth is a critical susceptibility window of the development^(32)^. Aside from this, children under 2 years have been reported to be at a high risk of Fe deficiency, which is a key risk factor for anaemia^(31)^. Additionally, most mothers, particularly the poor, may regard children under 2 years as being too young, thereby failing to initiate complementary feeding early enough in order to ensure that children receive the necessary micronutrients and reduce the risk of anaemia^(29)^.

In relation to micronutrient supplement consumption, iodised salt use was found to be associated with anaemia status. This iodine supplementation has been reported to improve Hb levels amongst children^(33)^. Just as was observed in stunting, children born to young mothers and non-educated mothers were found to be more probable of being anaemic^(34)^.

Place of residence and household wealth were found to be key risk factors for anaemia. Studies have confirmed that childhood anaemia is disproportionately concentrated in low socio-economic groups and rural areas^(31,35)^. Poor and rural households often have poor nutritional status and as were reported by Wegmuller *et al.* ^(36)^, anaemia and various micronutrient deficiencies (vitamin A, folate and Fe) seem to be more endemic in rural areas and poor households in Ghana.

Women who deliver at home are likely to have little access to pregnancy-related health services which include receiving essential services such as nutrition education and micronutrient supplementation which are crucial in improving the health status of their children^(37)^.

### Exploring the trend of risk factors of childhood stunting and anaemia

The overarching reason underlying the continual existence of childhood stunting and anaemia, in Ghana can be attributed to the pervading presence of the risk factors associated with these health outcomes.

For instance, the consistent association noted with respect to child’s age and gender in stunting and anaemia may be attributed to the fact that most nutritional policies implemented amongst children pay little attention and gender and age distributions of child malnutrition indicators. For instance, concerning age disparities, it has been reported that routine childhood growth monitoring, which is instrumental in the early identification of childhood malnutrition and provides the opportunity for the administration of micronutrient supplements such as vitamin A, has been reported to be significantly low amongst children between 2 and 5 years in Ghana^(38)^. Ghartey^(38)^ reported that most caregivers often consider the 0–2 years age interval as the critical period to attend growth monitoring sessions and immunisation. Sadly after this age interval, caregivers begin to pay little attention to these sessions, as they would rather prefer to engage in income generation ventures to help support their family^(38)^.

In Ghana, over the years, public health policies, strategies, and programmes have been initiated and implemented to address childhood stunting and anaemia; however, the majority of these initiatives focus on improving access to maternal and child health services such as hospital-based delivery, antenatal and postnatal visit, immunisation, vaccination, micronutrient supplementation (Fe and vitamin A supplementation), and diet fortification (iodised salt)^(13,39,40)^. Although these policies provide an excellent framework for improving access and utilisation of health services as well as addressing dietary-related factors, endemic challenges owing to socio-demographic inequalities often derail their impact. For instance, policies such as micronutrient supplementation (vitamin A, Fe and Iodine fortification) which were implemented within the later end of the 20th century are in urgent need of review and improved government funding^(38)^. These pitfalls have led to a widening of inequalities in the implementation of the policies, thereby resulting in the weakening of the desired effect expected by some of these policies. The resultant effect has been the increased risk observed amongst the risk groups as noted in the case of the effect of iodine status on anaemia.

Furthermore, to reduce the risk and occurrence of adverse birth outcomes such as low birth weight and childhood illnesses amongst children, policies such as the National Health Insurance Scheme (NHIS) have been rolled out to improve maternal healthcare services^(41)^. However, whilst this scheme was mandated to also help decrease socio-economic inequalities in healthcare utilisation within the country, studies have highlighted the existence of inequalities^(42,43)^. Fenny *et al.* ^(42)^ and Asamoah and Agardh^(43)^ have reported that these inequalities in maternal health services occur along the lines of the urban and rural divide and wealth and education statuses. These endemic inequalities might be a key factor in the continual occurrence of home deliveries in Ghana, which was observed to be significantly associated with either increased risk in both outcomes (stunting and anaemi) albeit with somewhat inconsistent trends.

In the case of low birth weight and childhood history of illness which were observed to be showing an increasing trend in relation to stunting and anaemia, respectively, improving maternal and child healthcare services may not be enough to address them properly. For instance, policies such as sleeping insecticide and vaccination which are crucial in preventing childhood infections seem to experience some setbacks in implementation. Aryeetey *et al.* ^(13)^ observed that although there was an increase in the ownership of insecticide-treated bed nets, between 2008 (42 %) and 2014 (68 %), only about a third of young children slept under these treated nets in 2014. Similarly, only about a third of young children received deworming medication in the past 6 months in 2014^(13)^. This situation could be a probable reason why effect estimates of child illness showed an increasing trend.

Child feeding indicators in Ghana have remained largely inconsistent, thus explaining the inconsistency in the effect estimates observed in this present study. According to Aryeetey *et al.* ^(13)^, there was no clear improvement in exclusive breast-feeding practice which is a key part of MDD between 2009 and 2018. While improvements were reported in 2008 (63 % 2008), there was however a drastic decline to about 43 % in 2017. Aryeetey *et al.* ^(13)^ again noted there has been a consistent decline in complementary feeding performance amongst children between 2009 and 2017 with the decline observed in both MDD and minimum acceptable diet rates in the last decade.

Maternal factors such as age and education are critical factors affecting both maternal and child health outcomes. As was observed in the study, younger mothers are more likely to have children who are malnourished. In Ghana, adolescent pregnancy has remained rife with very few changes observed in its prevalence^(44)^. Concerning maternal education, as highlighted in a multi-country study by Smith *et al.* ^(45)^, women of higher education status are more likely to have children with better nutritional status seeing that they have better nutritional status themselves and provide higher quality care to their children. In recent years, there has been progressive improvement in women’s education in Ghana^(46)^. Despite this progress, it must be highlighted there are still some key disparities based on various intersecting axes. Whilst at early-life and youthful ages, both males and females are almost parred in relation to educational attainment, the gap widens as one progresses into the adult and elderly populations^(47)^. As observed in this study, whilst stunting and anaemia were probable to occur amongst children of mothers with no education, the trends of the effect estimate differed for conditions. Although it is unclear why this differing trend was observed, it can be suggested that the inherent disparities in maternal education distribution across regions and wealth status could be a contributing factor^(47)^. Also, a large majority of the policies seem to focus on improving only basic education, which although helpful may not fully equip mothers with the requisite knowledge needed to strongly impact the nutritional status of their children.

In relation to household economic status, as per the Ghana Living Standards Report, poverty trends in Ghana between 1992 and 2013 reduced by more than half (from 56·5 % to 24·2 %), thus explaining the increase in the rich category as observed in the present study^(48)^. Cooke *et al.* ^(48)^ also noted that child poverty, which could be a key indicator of child health status, has also reduced significantly across the years. Afful *et al.* ^(49)^ noted that despite improvements in the economic growth rate between 1992 and 2013, this has not been complemented by a decline in inequality. This inequality has been reported to occur across various social stratification^(49)^, consequently leading to an increasing number of people living in extreme poverty. For instance, Cooke *et al.* ^(48)^ noted that the inequity in the ratio of children to adults living in poverty had risen substantially from the 1990s with about 15 % more children being likely to live in extreme poverty than adults^(48)^. This could explain the consistency in the effect estimates associated with poor households for both anaemia and stunting.

### Strengths and limitations

The present study provides evidence on the trend of the effect estimates of the risk factors associated with childhood stunting and anaemia. To the best of the authors’ knowledge, only one similar study has been conducted^(29)^. The aforementioned study only explored the risk factors of stunting and used only DHS datasets (2003, 2008 and 2014). This study, however, examined both stunting and anaemia and used secondary data from both the DHS and MICS which helps bridge the missing year gaps. Both surveys use nationally representative sample sizes and have high response rates, thus making the present study findings generalisable. Also as relates to regression model choice, the use of Poisson regression to generate PR offers some key advantages. As noted by Barros and Hirakata^(50)^, Poisson regression in cross-sectional studies provides more correct and consistent estimates than logistic regression, which is traditionally used in case–control studies. Besides this, it has been reported that when the outcome is interest is rare (less than 10 %) OR are similar to PR; however, when the outcome prevalence is not rare (greater than 10 %) as can be observed in this study, the OR often overestimates the PR^(51)^.

Despite these strengths, it must be noted that because the study relies on cross-sectional data, temporarity cannot be established. Aside from this whilst many risk factors could have been explored, however, to prevent overburdening of the regression model, such risk factors were logically dropped. To prevent multicollinearity between household environmental conditions (sanitation, water source and cooking fuel type) and household wealth, the former was dropped for the multivariate model. We, however, tried to address this by undertaking a mixed effect Poisson regression with the multiple adjusted models being considered to account for the dropped covariates.

### Conclusion

From this study, childhood stunting and anaemia were found to be significantly negatively associated with a wide array of socio-demographic (child age and gender, maternal age and education, place of residence, and household use of iodised salt) economic (household wealth) and health service (place of delivery and vaccination) factors. The trend of PR of the risk factors of the health outcomes reported was generally inconsistent. However, the most consistent risk factors associated with stunting and anaemia were child age and household wealth. To address these child malnutrition indicators, key consideration must be given to the early developmental stages of life. Also, child health policies must focus on addressing the key contextual factors of child malnutrition.
